# Cancer prevention: cervical cancer

**DOI:** 10.3332/ecancer.2019.952

**Published:** 2019-07-25

**Authors:** Mamsau Ngoma, Philippe Autier

**Affiliations:** 1 Ocean Road Cancer Institute, PO Box 5408, Dar Es Salaam, Tanzania; 2 International Prevention Research Institute, Espace Européen d'Ecully, Bâtiment G, Allée Claude Debussy, 69130 Ecully ouest Lyon, France

**Keywords:** cervical cancer, prevention, screening, HPV, vaccination

## Abstract

Cervical cancer is the fourth most common cancer in women, and the seventh overall, with an estimated 528,000 new cases and 266,000 deaths in 2012 [1]. Almost nine out of ten (87%) cervical cancer deaths occur in the less-developed regions of the world. The cervical cancer incidence significantly increases after 20 years of age and peaks at 50 years of age. Because cervical cancer mainly affects African women at a relatively young age, the socio-economic consequences are enormous. The human papillomavirus (HPV) is central to the development of cervical neoplasia and can be detected in 99.7% of cervical cancers. Hence primary prevention aims at reducing human papillomavirus (HPV) infection by HPV vaccine administration. Secondary prevention involves cervical cancer screening and management of precancerous lesions via either Pap smear, visual inspection with acetic acid (VIA) or with lugols iodine (VILI) or HPV testing for high-risk HPV types.

**Conclusion:**

Sub-Saharan countries still have a long way to go in controlling the high burden of cervical cancer. Effective prevention methods exist, such as HPV vaccination and screening, but their affordability and implementation remain challenging for most of these countries. Despite that, there is still light on the horizon, as the cost of HPV vaccines has been steadily decreasing and most African countries are using the more cost-effective methods of cervical cancer screening.

## Background

Cervical cancer is the fourth most common cancer in women, and the seventh overall, with an estimated 528,000 new cases and 266,000 deaths in 2012 [1]. Almost nine out of ten (87%) cervical cancer deaths occur in the less-developed regions. Although the registration of cancer cases and of cancer deaths is uncommon in Africa, it is estimated that about one-fifth of the world burden of cervical cancer cases and of deaths would occur in Africa [2]. The disease is unevenly distributed, with peak incidences of 30 cases and more per 100,000 women per year in the central and eastern part of the continent, and in countries surrounding the Guinea Gulf [3]. In contrast, the incidence would be less than 10 cases per 100,000 women per year in African countries located around the Mediterranean Sea.

## Socio-economic burden of cervical cancer

The cervical cancer incidence significantly increases after 20 years of age and peaks at 50 years of age. Because cervical cancer mainly affects African women at a relatively young age, the socio-economic consequences are enormous. So, women in sub-Saharan Africa lose more years to cervical cancer than to any other type of cancer. Many women with cervical cancer can no longer take care of their family, and their death leaves many orphans.

## The cause of cervical cancer

There are two major histologic types of cervical cancer, squamous cell carcinoma (about 75%) which mostly starts at the transformation zone of the ectocervix and adenocarcinoma (about 25%) which arises in the glandular columnar layer of the endocervix. The human papillomavirus (HPV) is central to the development of cervical neoplasia and can be detected in 99.7% of cervical cancers [4]. It is mostly caused by chronic infection with high-risk strains of HPV (mainly the subtypes 16 and 18). The HPV subtypes associated with squamous cancer are different from those associated with adenocarcinoma [5].

## Risk factors

HPV infection is essentially a sexually transmitted infection (STI), and therefore many risk factors for cervical cancer are those associated with a higher risk of STI occurrence. Factors that increase HPV acquisition or transmission, and promote the oncogenic effect of the virus are widespread in Africa. These are:

- young age at marriage or first sexual intercourse (i.e., before age 15 or 16 years),
- a high number of sexual partners,
- polygamy,
- a partner that has sexual intercourse with other partners,
- unprotected sexual intercourse (e.g., non or infrequent use of condoms, diaphragms or gels),
- a history of sexually transmitted infections or diseases,
- low education, deprivation,
- non-attendance or non-access to cervical cancer screening.

In addition to these factors, an impaired ability to clear HPV infection is probably the strongest risk factor for cervical cancer in Africa. Impairment of the immune system due to being infected with human immunodeficiency virus (HIV) is associated with persistent HPV infection which is the primary cause of cervical intra-epithelial neoplasia that may progress into an invasive cancer if left untreated. African HIV-positive women would have a 2 to 12-fold higher risk of intraepithelial neoplasia (CIN) lesions compared with HIV-negative women [6, 7]. Hence, HIV-infected women without overt AIDS, and women with AIDS, are at increased risk for the development of cervical cancer. Cervical cancer occurs at a younger age among HIV-infected women, and the disease tends to be more aggressive than in non-HIV-infected women [8].

## Primary prevention in cervical cancer

### Human papillomavirus vaccination

There are three different vaccines, which vary in the number of HPV types they contain and target, although not all are available in all locations:

- Quadrivalent HPV vaccine (Gardasil®) targets HPV types 6, 11, 16 and 18.
- 9-valent vaccine (Gardasil 9®) targets the same HPV types as the quadrivalent vaccine (6, 11, 16 and 18) as well as types 31, 33, 45, 52 and 58.
- Bivalent vaccine (Cervarix ®) targets HPV types 16 and 18.

The target group for vaccination recommended by the WHO are girls aged 9 to 14 years who have not become sexually active. This is because it has been shown that they have a better immune response to the vaccine than those in their late teens.

The choice of vaccine will depend on the availability and cost. Typically, two doses of HPV vaccine should be given at 0 and at 6 months but for those above 15 years require three doses, the last dose being at 12 months.

The vaccines prevent over 95% of HPV infections caused by HPV types 16 and 18 and may have some cross-protection against other less-common HPV types which cause cervical cancer [9]. HPV vaccination is effective in preventing cervical disease, including cervical intraepithelial neoplasia (CIN2 or 3) and adenocarcinoma in situ. Two of the vaccines also protect against HPV types 6 and 11 which cause anogenital warts. The vaccines work best if administered prior to exposure.

Data demonstrating the high effectiveness of HPV vaccination have been recently released for Scotland. In 2008, Scotland started a national immunisation programme against HPV using the bivalent vaccine. This programme was school-based and targeted girls aged between 12 and 13 with a catch-up programme until age 18. Uptake in the routinely immunised cohorts consistently exceeded 85% with 65% uptake in the catch-up cohort. Routine vaccination of girls aged 12–13 with the bivalent vaccine in Scotland has led to a dramatic reduction in pre-invasive cervical disease. Compared to un-vaccinated women born in 1988, vaccinated women born in 1995 and 1996 showed an 89% reduction (95% CI: 81%, 94%) in prevalent CIN Grade 3 or worse [10].

The safety of anti-HPV vaccination has received great attention. In 2014, the WHO stated that the two commercially available HPV vaccines ‘continued to have an excellent safety profile’ [11].

The primary prevention of cervical cancer largely overlaps with the primary prevention of STIs, including that of HIV. In Africa, many studies have been conducted on behaviours and methods associated with lower prevalence and transmission of STIs. Because STIs in general are associated with numbers of sex partners and unprotected sexual intercourse, prevention policies first focused on reductions of sex partners and on the provision of ‘barrier methods’, such as condoms. Information on the need to reduce high-risk sexual relationships has proven effective in Uganda where this policy has contributed to the control of HIV transmission [12]. Both circumcision and regular condom use are associated with reduced risk for oncogenic and overall HPV [13]. Nevertheless, the incidence of HPV infection among circumcised males and their partners remains high. And the high transmissibility of HPV infection suggests that primary prevention of STIs alone is not sufficient it should go hand in hand with HPV vaccination.

## Secondary cervical cancer prevention: cervical cancer screening

The goal of screening is to decrease the mortality associated with cervical cancer through detecting the disease when still at an early curable stage, or through detecting precursor lesions, i.e., the cervical CIN. The systematic removal of CIN lesion during screening also leads to reductions of the incidence of invasive cervical cancers of all stages.

Historically, visual inspection of the cervix without magnification was the first method of screening of the cervix. Currently, three different types of tests are promoted:

- Conventional Pap smear (or cytology) and liquid-based cytology
- Visual inspection with Acetic Acid (VIA) or with lugol iodine (VILI)
- HPV testing for high risk HPV types (e.g., types 16 and 18).

The randomised trials on cervical cancer screening have all been conducted in India, and have documented the efficacy of visual inspection, cytology screening and HPV-screening [18–21]. Numerous epidemiological studies have consistently documented that in countries where the resources exist to ensure high-quality and good coverage of the population, cytology screening contributes to decreasing the incidence of advanced-stage cancers and mortality associated with cervical cancer [22–24].

A priori, VIA or with VILI are the screening methods that would be the most cost-effective in African environments. VIA and VILI are performed in women who are 30 years up to 50 years. Women younger than 30 years should not undergo screening except for women known to be HIV-infected or living in a high HIV prevalence area.

A screening test followed in the same visit by treatment of positive results is referred to as a ‘screen and treat’ or ‘see and treat’ protocol. This approach is only possible with screening tests that produce immediate results (e.g., visual inspection, rapid-result HPV testing). The treatment methods mostly used are cryotherapy, loop electrosurgical excision procedure or cold-knife conisation.

There are also ‘Two-visit protocols’ which typically include a first visit with cervical cytology followed by a second visit with colposcopy and treatment based on the colposcopic examination. Two-visit protocols should not be used in populations where patients cannot afford (e.g., for economic or logistic reasons) more than one visit to outpatient clinics.

HPV testing detects strains of the virus that are associated with a high risk of cervical neoplasia. There is no commercially available test for detection of low-risk HPV strains. A specimen for HPV testing can be obtained using a Dacron swab or an endocervical brush, or some liquid-based cytology samples can also be used for HPV testing.

There are two types of available HPV tests:

- Tests that detect the presence or absence of any of 13 to 14 high-risk HPV subtypes that are associated with cervical cancer. These tests do not report which of the individual subtypes are present.
- Tests that perform HPV genotyping and report the presence or absence of HPV 16 or 18, such as Hybrid Capture II HPV testing

## Screening for cervical cancer in HIV-infected women and adolescents

Women who are infected with HIV should undergo cervical cancer screening twice in the first year after diagnosis of HIV infection and then annually, provided the test results are normal.

For women with two consecutive normal cytological examinations, the recommendation is that annual follow-up includes a thorough visual inspection of the anus, vulva, and vagina, as well as the cervix.

There is no consensus as to whether HPV testing should be performed routinely on HIV-infected women.

## Discussion

Several methods exist for cervical cancer prevention. All represent complex undertakings, incurring costs and the need of competent human resources.

Primary prevention based on condom use and circumcision are efficient methods. However, there is little spontaneous inclination to accept these methods and adequate information is necessary for disseminating the conviction that one can fight a deadly disease (and STIs in general) with these methods.

Countries contemplating the introduction of screening technologies should not overlook major issues encountered when planning screening activities. A first issue is the need to ensure therapy of screen-detected lesions. In this regard, visual inspection techniques might be the most cost-effective. Moreover, screening could be provided as a priority to HIV-infected women and to women with an STI.

Nationwide implementation of cytology screening or on HPV-detection is presently out of reach for most African countries. These screening technologies are costly and require the training of smear test readers, the shipment of tests, and more than one encounter with screened women, which may be difficult in many settings.

Nationwide HPV vaccination programmes are facing economic constraints, mainly the cost of vaccines which remain a barrier for countries where the health budget is only a few euros per capita per year. However, costs of HPV vaccines tend to steadily decrease, and thus affordability is rising on the horizon.

A recurrent question is whether priority should be given to screening or to HPV vaccination policies. Where screening is rare (i.e., most of Africa), probably anti-HPV prophylaxis should be considered first, as this policy is likely to drastically reduce the burden of HPV-related genital neoplasia on the long term. One way to contemplate screening versus vaccination issues is to conduct cost-effectiveness studies in countries where cervical cancer control is on the agenda. Up to now, most economic studies have focused on HPV vaccination considered alone, or in combination with screening like in South-Africa [25–28]. Future economic studies should compare the marginal cost-effectiveness of HPV vaccination against screening using, say, visual inspection according to a one-step strategy. These studies could also address specific issues like young women with HIV infection, for which screening starting at an early age (say around 20 instead of 30 years of age) could remain a valid strategy. Such studies have the potential to provide an exhaustive overview of needs and constrains for each approach in resource-limited settings, and provide metrics for comparing them and make informed choices.

## Conclusion

Sub-Saharan countries have still a long way to go for controlling the high burden of cervical cancer. Effective prevention methods exist, such as screening, HPV vaccination and safe sex behaviours, but their affordability and implementation remain challenging for most of these countries. The perspective of steadily decreasing costs of HPV vaccines is an invitation for examining cost-effective ways to plan vaccination programmes, and consider whether vaccination should not be given the priority.

## Conflicts of interest

None to disclose.

## Funding statement

This work was part of academic duties. No external funding source supported this work.
